# Supplementary material for: Combining Engineered U1 snRNA and Antisense Oligonucleotides to Improve the Treatment of a BBS1 Splice Site Mutation
Source: Mol Ther Nucleic Acids. 2019 Aug 16;18:123–30. doi: 10.1016/j.omtn.2019.08.014 (PMC6796732; doi:10.1016/j.omtn.2019.08.014)
Supplement: Document S1. Figure S1 [file mmc1.pdf]

OMTN, Volume *18*

Supplemental Information

Combining Engineered U1 snRNA and Antisense Oligonucleotides to Improve the Treatment of a *BBS1* Splice Site Mutation

Saskia Breuel, Mariann Vorm, Anja U. Bräuer, Marta Owczarek-Lipska, and John Neidhardt

Supplemental Material

Combining designed U1 snRNA and antisense oligonucleotides to improve the treatment of a *BBS1* splice site mutation.

Saskia Breuel[1], Mariann Vorm[1], Anja U. Bräuer[2,3], Marta Owczarek-Lipska[1], John Neidhardt[1,3,4,]*

Figure S1:

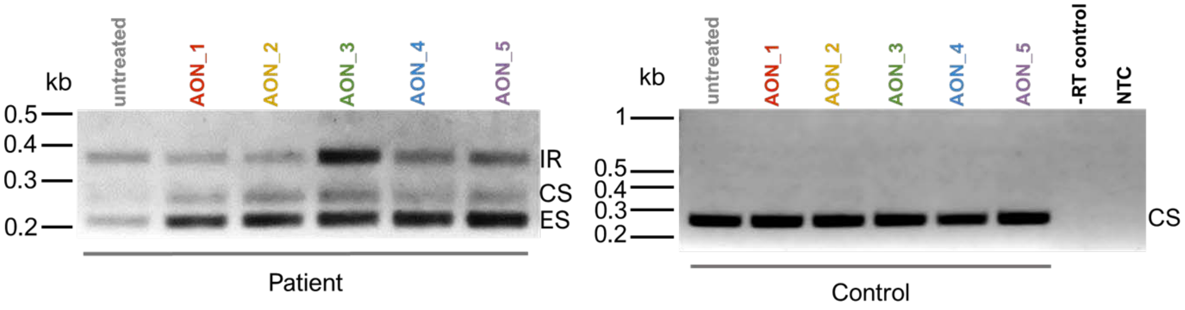

Fig. S1: Verification of AON effects during the apoptosis assay. Cells were lyzed and RT-PCR reactions were performed after the apoptosis assays. AON concentrations were 36 nM. The efficacy of the tested AONs to reduce intron 5 retention in the patient samples resembles the results shown in Fig. 3. The AON treatment did not induce splice defects in control cells. NTC: none template control; –RT control: PCR control reaction using a template with RNA but no reverse transcriptase was added during cDNA-synthesis. CS: correctly spliced transcript, ES: exon skipping, IR: intron retention, kb: kilo bases.
