# Supplementary material for: Combining Engineered U1 snRNA and Antisense Oligonucleotides to Improve the Treatment of a BBS1 Splice Site Mutation
Source: Mol Ther Nucleic Acids. 2019 Aug 16;18:123–30. doi: 10.1016/j.omtn.2019.08.014 (PMC6796732; doi:10.1016/j.omtn.2019.08.014)
Supplement: Document S2. Article plus Supplemental Information [file mmc2.pdf]

Molecular Therapy
Nucleic Acids
Original Article

Combining Engineered U1 snRNA and Antisense Oligonucleotides to Improve the Treatment of a *BBS1* Splice Site Mutation

Saskia Breuel,[1] Mariann Vorm,[1] Anja U. Bräuer,[2,3] Marta Owczarek-Lipska,[1] and John Neidhardt[1,3,4]

[1]Human Genetics, Faculty of Medicine and Health Sciences, University of Oldenburg, Oldenburg, Germany; [2]Anatomy, Faculty of Medicine and Health Sciences, University of Oldenburg, Oldenburg, Germany; [3]Research Center Neurosensory Science, University of Oldenburg, Germany; [4]Joint research training group of the Faculty of Medicine and Health Sciences, University of Oldenburg, Germany and the University Medical Center Groningen, Groningen, Netherlands

Manipulation of pre-mRNA processing is a promising approach toward overcoming disease-causing mutations and treating human diseases. We show that a combined treatment applying two splice-manipulating technologies improves therapeutic efficacies to correct mutation-induced splice defects. Previously, we identified a family affected by retinitis pigmentosa caused by the homozygous *BBS1* splice donor site mutation c.479G > A. The mutation leads to both exon 5 skipping and intron 5 retention. We developed a therapeutic approach applying lentivirus-mediated gene delivery of engineered U1 small nuclear RNA (U1), which resulted in increased levels of correctly spliced *BBS1*. Herein, we show that the therapeutic effect of the engineered U1 efficiently reverted exon skipping but failed to reduce the intron retention. To complement the engineered U1 treatment, we identified four different antisense oligonucleotides (AONs) that block intron 5 retention in *BBS1* transcripts. A treatment using engineered U1 in combination with AONs showed the highest therapeutic efficacy and increased the amount of correctly spliced *BBS1* transcripts. We did not detect elevated levels of apoptotic cell death in AON-treated cell lines. In conclusion, engineered U1 or AONs provide efficient therapies with complementary effects and can be combined to increase efficacy of therapeutic approaches to correct splice defects.

INTRODUCTION

The Bardet-Biedl syndrome (BBS, also known as Laurence-Moon-Bardet-Biedl syndrome) constitutes a rare autosomal recessive disease that affects several organs. This pleiotropic disease is characterized by retinal degeneration, kidney dysplasia and dysfunction, polydactyly, obesity, hypogonadism, and learning difficulties. Clinical variability is frequently observed in BBS patients. Secondary BBS features may, among others, include developmental delay, diabetes mellitus, dental anomalies, congenital heart diseases, and olfactory deficits.

The prevalence of BBS is estimated to be 1:100,000 in North America and Europe, but may increase in subpopulations or isolated communities (1:18,000 in Newfoundland, 1:13,500 in Bedouin communities, and 1:4,000 on the Faroe islands).[1–3] Heterogeneity exists among *BBS* genes and until now, 21 genes are known to be associated with BBS.[4] These genes include *BBS1–BBS20* and *NPHP1*. Biallelic mutations in these genes account for approximately 80% of the cases. Interestingly, several of these genes may also cause other diseases than BBS, including McKusick-Kaufmann, Alström, and Meckel Gruber syndromes. *BBS1*, *BBS2*, and *BBS10* are the most frequently mutated genes in BBS-affected patients.[4,5]

All BBS-associated gene products influence ciliary properties. Cilia are finger-like protrusions from the cell surface that can be envisioned as cellular antennae influencing several signaling cascades. Indeed, BBS belongs to a group of disorders called ciliopathies in which ciliary function is disturbed. Because cilia are involved in developmental processes and are expressed on many cells of the human body, it is not surprising that the pleiotropic nature of the disease often manifests in BBS patients.

The gene *BBS1* was found to cause BBS in 2002.[6] It contains 17 exons and is located at chromosome 11 spanning approximately 23 kb. The mutation p.Met390Arg is frequently found in *BBS1* and the knockin mouse model of this particular mutation resembles phenotypic aspects of the human disease.[7] Overall, more than 75 mutations have been described in *BBS1*, many of which represent amino acid changes, splicing mutations, or small deletions (for reference see HGMD database, http://www.hgmd.cf.ac.uk/ac/index.php).

The genetic and clinical heterogeneity in BBS raises challenges in the treatment of the patients.[5] To date, symptomatic treatment is the only therapeutic option, often involving several medical expertise. Novel therapeutic approaches are needed to overcome the deleterious consequences of *BBS* gene mutations, including the development of genetic therapies that apply the technologies of gene replacement,

Received 1 March 2019; accepted 1 August 2019;
https://doi.org/10.1016/j.omtn.2019.08.014.

Correspondence: Prof. Dr. John Neidhardt, PhD, Human Genetics, Faculty of Medicine and Health Sciences, University of Oldenburg, Ammerländer Heerstrasse 114-118, 26129 Oldenburg, Germany.
E-mail: john.neidhardt@uol.de

This is an open access article under the CC BY license (http://creativecommons.org/licenses/by/4.0/).

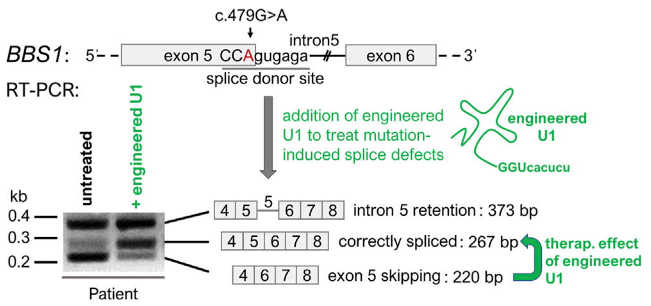

Figure 1. Engineered U1 Was Able to Reduce Exon Skipping, But Failed to Prevent Intron Retention

Schematic drawings show parts of the pre-mRNA of exon 5-intron 5-exon 6 of *BBS1* and summarize the observed splice variants of *BBS1*. The splice donor site is underlined. Capital letters denote exonic bases and small letters represent intronic bases. The *BBS1* gene mutation c.479G > A (red letter) locates to the last base of exon 5 and causes both exon 5 skipping (220 bp) and intron 5 retention (373 bp) in *BBS1* transcripts. Residual levels of correctly spliced *BBS1* transcripts (267 bp) were detectable in untreated patient-derived fibroblast cells using RT-PCR analyses and agarose gel electrophoresis. U1 snRNA was engineered to show full complementarity to the mutated splice donor site in *BBS1* (green drawing, complementary base pairs: GGUcacucu). Upon treatment of the patient-derived fibroblasts with the engineered U1, exon 5 skipping can be reverted and increased amounts of correctly spliced *BBS1* transcripts were detected (green arrow). In contrast, the intron 5 retention remained unchanged by the U1 treatment. kb, kilo bases; bp, base pairs.

CRIPSR-Cas, antisense oligonucleotides (AON), or engineered U1 small nuclear RNA (U1) splice factors. All of these technologies have been demonstrated be able to efficiently correct mutation-induced defects of a target gene.

Previously, we described a family with a homozygous mutation in *BBS1* leading to a mild phenotype of retinitis pigmentosa.[8] This mutation affects the splice donor site of exon 5 and causes both exon 5 skipping and intron 5 retention. We have now developed a novel therapeutic approach that applies a combination of AONs (to block the intron 5 retention) and an engineered U1 (to correct the exon 5 skipping) with the aim to synergistically improve treatment efficacies.

RESULTS

We previously showed that the homozygous *BBS1* mutation c.479G > A leads to both exon 5 skipping and intron 5 retention within the *BBS1* transcript.[8] We also demonstrated that U1 can be engineered to partially correct mutation-induced splice defects in *BBS1*. As summarized in Figure 1, we verified the previous results and confirmed that engineered U1 (showing full complementarity to the mutated splice donor site in *BBS1*) efficiently reduced the exon skipping events and simultaneously increased the amount of correctly spliced *BBS1* transcripts. In contrast to the therapeutic effect on exon skipping, the engineered U1 treatment failed to reduce the intron 5 retention (Figure 1). Using Sanger sequencing, we confirmed skipping of exon 5 in the lower band (220 bp), the mutation in the correctly spliced *BBS1* transcripts (267 bp), and the retention of intron 5 in the upper band (373 bp).

AONs are small oligonucleotides typically around 20 bp in length. These small pieces of RNA or DNA can interfere with the splicing mechanism by complementary base pairing with a target sequence, a process that often hinders splice inhibitors and/or enhancers to influence splicing of a target transcript. Thereby, AONs show the potential to correct mis-spliced transcripts.[9–14]

We hypothesized that intronic splice enhancers may bind to intron 5 of the *BBS1* pre-mRNA transcript and thus, promote intron 5 retention as a consequence of the mutated splice donor site (Figure 2A). Blocking the binding sides of intronic splice enhancers might even correct the splice defect seen in the patient-derived cell line (Figure 2A). To test this hypothesis, we applied an AON generated to specifically bind to intron 5 of *BBS1* and performed a RT-PCR analysis. We found that this AON (AON_1) strongly decreases the occurrence of the intron 5 retention band in fibroblasts derived from *BBS1* patients (Figure 2B). Furthermore, we observed that the AON_1 not only reduced intron retention, but also seemed to facilitate exon skipping of *BBS1* exon 5 (Figure 2B). It was less clear whether the AON treatment also supported the generation of normally spliced *BBS1* transcripts in the patient-derived cell line.

In an attempt to optimize the AON_1 effect (Figure 2B), we generated five additional AONs (AON_2 to AON_6) and compared their efficacy to reduce the ratio between the intron retaining transcript and the other two detected *BBS1* transcripts in patient-derived cells homozygous for the *BBS1* c.479G > A mutation (Figures 3A and 3B). Selection of all six AONs was supported by predicted splice factor binding sites (ESEfinder: http://krainer01.cshl.edu/tools/ESE2/). The selected AONs showed binding sites within 75 bp from the exon-intron boundary of *BBS1* exon 5 and were up to 22 bp long (Figure 3A). We tested three different AON concentrations (AONs were applied to the culture medium without transfection reagent) and found that AON_1, AON_2, AON_4, and AON_5 significantly reduced the intron 5 retention seen in the patient cell line. Figure 3B shows a representative example of these analyses (of note, due to limited loading capacity Figure 3B shows AON_1 only in the highest concentration tested). In accordance with the literature, we applied the AONs for 96 h before cell lysis and RT-PCR analyses.[9,15] Concluding from these results, none of the other AONs were clearly more efficient in reducing the intron 5 retention in the *BBS1* patient cell line compared to AON_1. AON_1 and AON_2 seemed to show similar capacities to interfere with the intron 5 retention therefore we decided to continue with AON_1 in the following experiments.

Furthermore, none of the AONs induced mis-splicing of *BBS1* transcripts in the control fibroblast cell line (Figure 3C) suggesting that the treatment with AONs is not interfering with normal splicing of *BBS1*. To further evaluate possible side effects of the AONs, we performed an apoptosis assay with three different AON concentrations (9 nM, 18 nM, and 36 nM). The efficacy of the AON to block the intron retention was verified after the apoptosis assay (Figure S1). None of the tested conditions showed a marked increase in

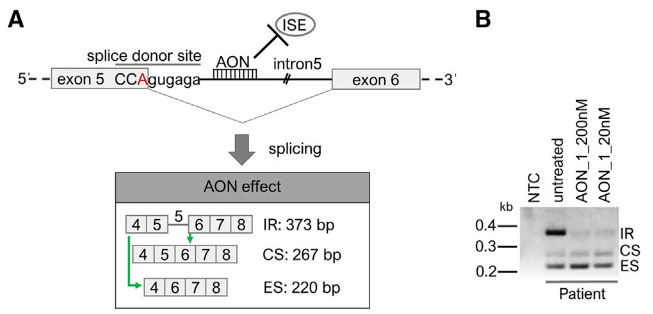

Figure 2. AON Treatment of Patient-Derived Fibroblasts

(A) Schematic drawing of the hypothesized action of AONs on the transcript processing of *BBS1*. AONs may interfere with binding of intronic splice enhancers (ISEs), the consequence of which would be increased levels of both exon 5 skipping and normal splicing of the mutated *BBS1* pre-mRNA transcript. Green arrows show the hypothesized effect of the AON. The red letter A marks the mutated base pair in *BBS1*. (B) The patient-derived fibroblast cell line was treated with AON_1 in two concentrations (20 nM and 200 nM). The treatment resulted in reduced intron 5 retention and increased exon 5 skipping. Whether the amount of correctly spliced *BBS1* transcripts was also increased upon the AON_1 treatment was less clear. The AON was applied to the patient-derived cells for 24 h, the cells were harvested, RNA was isolated, and RT-PCR was performed. CS, correctly spliced transcript; ES, exon skipping; IR, intron retention; kb, kilo bases.

apoptotic cell death (Figure 4), a finding that supports the notion that AONs show little side effects within the applied concentrations.

Both of the two treatment approaches tested herein (either using AONs or engineered U1) showed therapeutic potential to reduce splice defects in the patient-derived cell line homozygous for the *BBS1* c.479G > A mutation. The AON treatment clearly reduced the mutation-induced intron 5 retention, whereas engineered U1 was efficient to revert the exon 5 skipping. We asked the question whether the combination of the two promising approaches would synergistically improve the efficacy of the splice correction in the patient-derived BBS1 cell line (Figure 5A).

Therefore, we treated the patient-derived fibroblasts simultaneously with both the AON_1 and the engineered U1. We found that this treatment improved the efficacy of the splice correction and increased the amount of correctly spliced *BBS1* transcripts compared to single treatments (Figures 5B and 5C). RT-PCR reactions were performed under highly comparable conditions. Semiquantitative measurements of band intensities documented significant differences between the untreated and treated samples (Figure 5C). We compared single and combined treatments (Figures 5B and 5C) and found that the combined treatment generated significantly more of the correctly spliced *BBS1* transcript than the single U1 treatment. Furthermore, we tested the dose dependency of the therapeutic effect by modulating the concentration of AON_1 while keeping the engineered U1 treatment constant (Figure 5D). The results suggested that higher AON_1 concentrations were beneficial for the treatment (Figure 5D).

DISCUSSION

Therapeutic applications to treat genetic diseases develop rapidly. The urgent need to find efficient and safe approaches to overcome the deleterious consequences of genetic disorders is driving the development of novel gene therapies. A combination of well-established approaches is an option to further improve limited efficacies of the existing gene therapies. In the present study, we demonstrate that the combination of therapeutic applications (AONs and engineered U1) may complement the treatment of splice defects and improve the efficacy compared to single treatments.

Splicing is a complex process of pre-mRNA maturation that involves hundreds of interaction partners.[16,17] Despite these complexities, the basic principles of splicing are well understood and suggest that several weak interactions between pre-mRNA and splice factors are required to ensure both high flexibility and precision during splicing.[18] The deeper understanding of the splicing mechanism has also facilitated the development of technologies to manipulate splicing processes in order to treat disease-relevant splice defects.[19,20] Around 20% of the mutations identified in disease-associated genes interfere with splicing, an observation that is almost independent of the disease gene.[21–23] The effects of mutations on splicing are multiple and may include exon skipping and intron retention, as well as cryptic splice site activation.[24,25] We and others have shown that the U1-based correction of splice defects represents a promising therapeutic approach. Highly efficient correction of splice defects was demonstrated using mutation-adapted (engineered) U1, either performing splice assays in cultured cells or even treating endogenously expressed transcripts in patient-derived cell lines.[8,26–33] Especially, mutation-induced exon skipping events were successfully treated. Importantly, the application of engineered U1 in cell lines did not indicate obvious side effects.[28] These studies further initiated the application of the U1-based treatment to mini-gene-based splice defects *in vivo*.[34] Together, approaches applying mutation-adapted (engineered) U1 provide a promising therapeutic technology to correct splice defects.

Of note, U1-based approaches may be combined with a second engineered U snRNA that is adapted to the same mutation, demonstrating that not only therapeutic approaches applying engineered U1 alone can be used to ameliorate splice defects. Later in the splicing cycle, U6 snRNA (U6) binds to the splice donor site and replaces U1. We previously found that engineered U1 in combination with engineered U6 lead to improved splice corrections. These treatment approaches applied two mutation-adapted snRNAs, a combination that was more efficient in correcting exon-skipping events than U1 alone.[35]

The experiments performed herein further suggest that intron retention is not efficiently treated using the engineered U1 technology. In order to overcome these limitations, we tested several AONs to prevent the intron 5 retention induced by the c.479G > A mutation in *BBS1*. Out of six tested AONs, four were able to efficiently inhibit intron 5 retention and thus showed therapeutic potential. Other studies reported similar efficacies of AONs to block falsely spliced

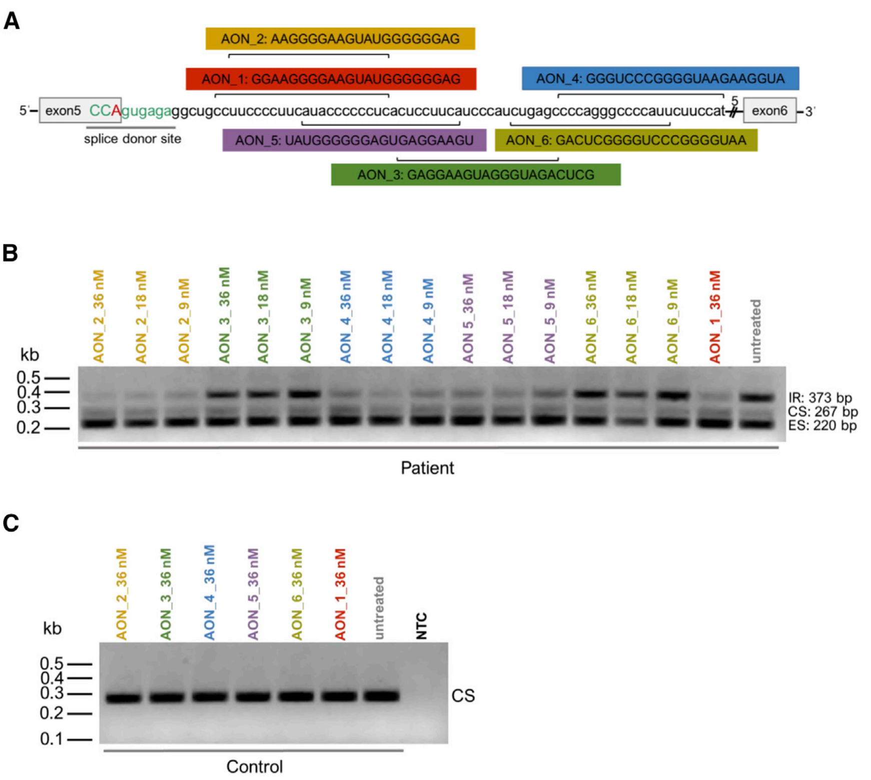

Figure 3. Comparison of Six Different AONs

(A) Schematic drawings show the binding sites of six AONs that bind to different sequences within intron 5 of *BBS1*. The mutated base pair (red letter) is located in the splice donor site of *BBS1* (light green letters). (B) Efficacies to block the intron retention in *BBS1* patient-derived fibroblasts were compared between six AONs (AON_1 to AON_6). Therefore, different concentrations of the AONs (9 nM, 18 nM, 36 nM) were incubated for 96 h. Results of the RT-PCR showed that AON_1, AON_2, AON_4, and AON_5 were efficient to block intron retention, whereas AON_3 and AON_6 showed no effect. The size of the different splice products is shown. (C) Control fibroblasts were incubated with AON_1 to AON_6 (36 nM) for 96 h to test for side effects. No splice alteration was detected. NTC, none template control; Patient, fibroblasts homozygous for the *BBS1* mutation c.479G > A; Control, fibroblasts with *BBS1* reference alleles. CS, correctly spliced transcript; ES, exon skipping; IR, intron retention; kb, kilo bases.

transcripts.[10,12,15,36,37] Nevertheless, it seems surprising that over 50% of the selected AONs tested in this study (binding sites varied over a stretch of approximately 60 bp) efficiently blocked the mis-spliced *BBS1* transcripts. This suggests that *BBS1* intron 5 requires several interaction partners to be spliced. Because AON_1, AON_2, and AON_5 show overlapping binding sites, we speculate that intron 5 of *BBS1* requires interaction partners and/or complexes in this region. A second binding complex can be expected in the binding site of AON_4 located further downstream in the intron 5 of *BBS1*.

The AONs tested in this study did not show obvious side effects. We did not find indications for increased apoptosis induced by the AONs. Nevertheless, another study has indicated decreased cell viability following treatment approaches in fibroblasts using higher AON concentrations for a different target gene.[9] Although AON treatment approaches are widely considered to be safe approaches, it cannot be excluded that transfection reagents and AON chemistry and concentration, as well as off-target binding, due to specific AON sequences, incubation times, and target gene selection determine the risk for AON-mediated side effects.

Mutations detected in disease-associated genes are often predicted to affect the encoded protein, although the impact of mutations on the protein level is not frequently verified by experiments. Consequently, the interference of mutations on splicing processes may be misjudged or even overlooked. We have previously identified the *BBS1* mutation c.479G > A, which was only predicted to lead to the amino acid exchange p.R160Q. In contrast, we showed that the mutation predominantly leads to splice defects in *BBS1* pre-mRNA transcripts. Residual levels of the normal *BBS1* transcript were detected, which likely lead to reduced levels of BBS1 proteins in patient cells and in turn might contribute to a milder phenotype observed in patients. Indeed, a milder phenotype was observed in the *BBS1*-affected patients studied herein. In contrast to the spectrum of phenotypes typically observed in BBS patients, we solely found a retinal dystrophy without any further signs of other BBS features.[8] The hypomorphic nature of the mutation was supported by stainings of cilia in patient-derived nose epithelial cells that did not present with obvious ciliary defects.[8] In contrast to our findings, Davis et al.[7] showed that the *Bbs1* knockin mouse model of the prominent p.M390R mutation interferes with ciliary properties. Similarly, other BBS-associated genes were reported to influence the length of cilia in patient-derived fibroblasts.[10,38] In conclusion, our observations of the hypomorphic nature of the *BBS1* c.479G > A mutation provided a molecular explanation for the exceptionally mild phenotype found in the patients studied herein and have implications toward understanding the enormous phenotypic spectrum seen in BBS patients.

MATERIALS AND METHODS

Ethical Statement

Research was carried out conforming to the tenets of Declaration of Helsinki. The collection of human skin biopsies and the use of human dermal fibroblasts were approved by the local ethics committees (Hannover Medical School, Germany [2576-2015] and Faculty of Medicine and Health Sciences at the Carl-von-Ossietzky University Oldenburg, Germany [2018-097]). Written informed consent was obtained from patients and control individuals participating in the study.

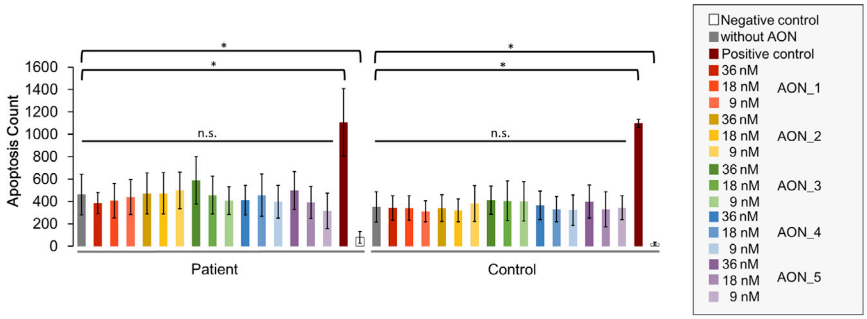

Figure 4. Evaluation of Side Effects Induced by AONs

Apoptosis assays were performed to evaluate side effects induced by the AONs. Five AONs (AON_1 to AON_5) were tested in three concentrations (9 nM, 18 nM, 36 nM) and were incubated for 96 h. Apoptosis assays were performed twice with independently cultured fibroblasts including four technical replicates. For capacity reasons in the apoptosis assay, as well as due to the fact that AON_6 was not efficient in reducing intron 5 retention, we did not include AON_6 in the assay. The graph shows the apoptosis count detected in patient and control fibroblasts (mean ± SD) after the treatment. Neither in patient-derived fibroblasts nor in controls did we detect a significant (n.s.) increase in apoptotic cell death. Significant differences (*) were detected between untreated cells (negative control) and cells that received the assay components without including an AON (without AON). The positive control received Camptothecin to induce apoptosis.

Design of AONs

We used the ESE finder 3.0 program (http://rulai.cshl.edu/cgi-bin/tools/ESE3/esefinder.cgi?process=home) and the RegRNA program (http://regrna.mbc.nctu.edu.tw/html/prediction.html) to predict potential binding sites for RNA binding proteins in intron 5 of *BBS1*. We designed six different AONs complementary to parts of *BBS1* intron 5 (BBS1-AON_1: 5′-GAGGGGGGUAUGAAGGGGAAGG-3′; BBS1-AON_2: 5′-GAGGGGGGUAUGAAGGGGAA-3′; BBS1-AON_3: 5′-GCUCAGAUGGGAUGAAGGAG-3′; BBS1-AON_4: 5′-AUGGAAGAAUGGGGCCCUGGG-3′; BBS1-AON_5: 5′-UGAAGGAGUGAGGGGGGUAUG-3′; BBS1-AON_6: 5′-AAUGGGGCCCUGGGGCUCAG-3′). Every AON was modified with a phosphorothioate backbone to ensure uptake and stability and a 2′-O-methyl group to further enhance stability.[39–43] Synthesis of AONs was performed by Eurogentec (Cologne, Germany) and AONs were dissolved in phosphate-buffered saline.

Cell Culture of Patient-Derived Fibroblasts and Treatment with AONs

Human dermal fibroblasts from patient and controls were cultured in Minimum Essential Medium (Biowest, Renningen, Germany), containing 20% fetal calf serum (Biowest), 1.4% L-glutamine (Biowest), and 1% Antibiotic-Antimycotic (Biowest) at 37°C and 5% CO_2. One day before treatment, 1.8–2.5 × 10^5 cells per well were seeded either in a twelve-well or six-well plate. One of six AONs was added directly to the medium without using any transfection reagent in different concentrations. Concentrations varied between 200 nM, 36 nM, 20 nM, 18 nM, and 9 nM. After 20–96 h of incubation at 37°C, the cells were washed with 1 mL of 1−× phosphate-buffered saline (Biowest), lyzed with 350 μl of lysis buffer RA1 (Macherey and Nagel, Düren, Germany), and supplemented with 3.5 μl of β-Mercaptoethanol (β-ME; Serva, Heidelberg, Germany). Lysates were harvested by pipetting up and down several times, as well as by scraping with a pipette tip.

RNA Isolation and RT-PCR

Total RNA from fibroblast lysates was purified using the Nucleospin RNA isolation kit according to the manufacturer's instructions (Macherey and Nagel). Synthesis of first-strand cDNA was performed with 180–800 ng RNA and random primers (Metabion, Planegg/Steinkirchen, Germany), according to the manufacturer's protocol, with the exception that we used 0.5 μl Superscript III (Supercript III; Invitrogen, Schwerte, Germany) per reaction. For each sample in an experiment, the same amount of RNA (normalized to the sample with the lowest RNA concentration) was used for cDNA synthesis. We used 1 μl of the cDNA reaction of each sample to allow direct comparison of RT-PCR results within one experiment. The *BBS1* transcript was amplified with the HotFire Taq Polymerase (Solis Biodyne, Tartu, Estonia) using primers located in exon 4 and exon 8 (BBS1-ex4 dn: 5′-GCCCCAATTGCCTCCAAATCCT-3′ and BBS1-ex8up: 5′-GCATCCTCGTCAGCCAGGTTCTTC-3′). PCR products were analyzed on 2% agarose gels.

Apoptosis Assay

Possible toxic effects of the different AONs were analyzed applying an apoptosis assay. A day before treatment, fibroblasts derived from patients and controls were seeded into a 96-well plate (2,250 cells per well). For apoptosis detection, cells were treated with IncuCyte Caspase-3/7 Green Reagent (Essen Bioscience, Ann Arbor, MI, USA) with a final concentration of 5 μM. Cells of one well treated with 400 nM Camptothecin (Biomol, Hamburg, Germany) served as a positive control. IncuCyte Caspase-3/7 Green Reagent was diluted in MEM and Camptothecin was diluted in DMSO (Roth, Karlsruhe, Germany) as recommended by manufacturer's protocol (Essen Bioscience and Biomol). We tested AON_1 to AON_5 in three different concentrations (36 nM, 18 nM, 9 nM). Untreated fibroblasts served as negative control. The plates were incubated for 96 h in an IncuCyte S3 Live-Cell Analysis System installed in an incubator at 37°C and 5% CO_2. Whole-well images were collected after 96 h in phase-contrast and green fluorescence with a 4× objective. Immediately after 96 h, cells were washed with 100 μl of 1× phosphate-buffered saline (Biowest), lyzed with 2 μl of β-ME (Serva) in 100 μl of lysis buffer RA1 (Macherey and Nagel), and stored in −80°C until RT-PCR experiments were performed. The assay was replicated twice with independently cultured cell lines and each experiment included two technical replicates. Quantification of apoptotic counts was performed with the IncuCyte S3 Software.

The AON efficiency during the apoptosis assay was verified by RT-PCR. RNA was extracted from the cell lysates using the Nucleospin RNA XS isolation kit (Macherey and Nagel), followed by cDNA synthesis and RT-PCR with 40 ng cDNA per reaction as described above.

Treatment with Engineered U1 snRNA and/or AONs

Productions of engineered U1 and wild-type U1 in lentiviral shuttles were performed as previously described.[8,28] In brief, HpaI restriction sites of the lentiviral plasmid p.RRLSIN.cPPT.SFFV/GFP.WPRE[44,45] were used for cloning of the human U1 small nuclear RNA cassette.[46] U1 was fully adapted to the mutated splice donor site (exon 5) of *BBS1* using site-directed mutagenesis.[27] HEK293T cells were cultured in DMEM (Biowest), supplemented with 10% fetal calf serum (Biowest), 1% L-glutamine (Biowest), and 1% penicillin and streptomycin (Biowest) at 37°C and 5% CO_2. We worked with a highly standardized protocol for lentivirus production: We seeded 7×10^6 HEK293T cells in a 75 cm^2 flask, followed by transfection of HEK293T cells with the two packaging plasmids pSPAX2 (19.5 μg) and pMD2.G (6 μg) and the expression plasmid containing the fully adapted U1 cassette (36 μg) using branched polyethyleneimine (75 μg) (Sigma-Aldrich, Munich, Germany). DMEM was replaced by MEM after 8. Medium containing the lentiviral shuttles was collected twice: after 20 h, changed to new MEM and harvested after 20 h again. The lentiviral containing medium was stored at 4°C until use and was pooled before transduction of fibroblasts. For transduction of fibroblasts, the virus was added to $2–2.5 \times 10^5$ cells in either a twelve-well or six-well plate. As an indicator of a successful lentiviral transduction, the fibroblasts were regularly checked for eGFP expression using fluorescence microscopy (Axio Vert.A1 microscope, Carl ZEISS AG, Oberkochen, Germany).

After 24 h incubation with the lentiviral shuttles, BBS1-AON_1 was added in different concentrations (36 nM, 30 nM, 26 nM, 13 nM, 6 nM) to verify the dose dependency of the treatment. Cells were harvested after 2, 3, or 4 days after the AON was added to the medium. Results were analyzed with the RT-PCR described above. Products

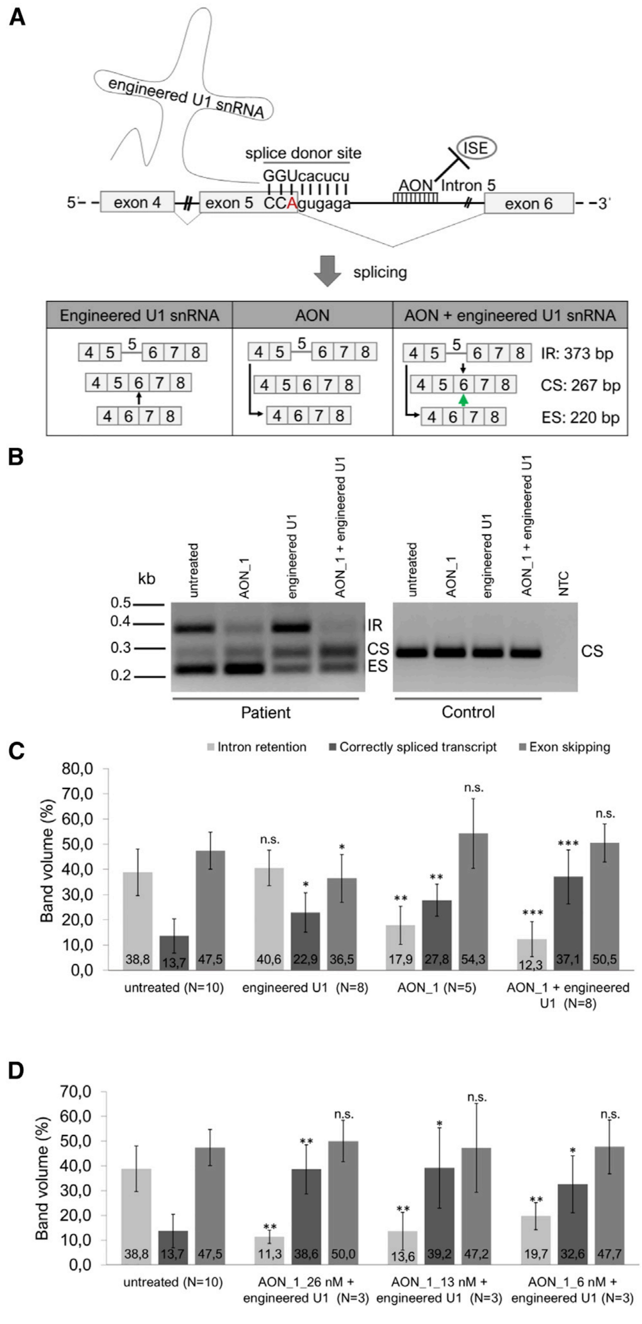

Figure 5. Combining the Treatment of AON and Engineered U1 Improved the Therapeutic Effect

(A) Schematic drawing of the therapeutic approach combining both engineered U1 and AON_1. Engineered U1 binds to the splice donor site and facilitates the recognition of *BBS1* exon 5. In contrast, the AON blocks the recognition of intron 5 during splicing. Compared to the single treatments, the combined treatment of engineered U1 and AONs further increased the level of correctly spliced *BBS1* transcripts. The green arrow highlights the effect of the combined treatment. (B) RT-PCR analysis of the combined AON_1 and engineered U1 treatment in patient-derived and control cell lines. Whereas the single treatment with AON_1 reduced the intron 5 retention, engineered U1 reduced the exon 5 skipping. A combination of both agents showed a synergistic effect and increased the amount of the correctly spliced *BBS1* transcripts. In the control cell line, splice defects were not induced by AON_1, engineered U1, or the combination of both approaches. Only correctly spliced *BBS1* transcripts were detected in the control cell line. (C and D) Semi-quantitative evaluation of band intensities of RT-PCR analyses performed under highly comparable and standardized conditions. (C) Treatments of engineered U1, AON_1, and the combination of both engineered U1 and AON_1, were compared to untreated conditions of the patient-derived fibroblasts. The most significant increase of correctly spliced *BBS1* transcripts was detected following the combined treatment with both engineered U1 and AON_1. (D) Different concentrations of AON_1 were tested for their efficacy to prevent intron 5 retention. With the highest concentration of AON_1, we detected the highest significant increase of the correctly spliced *BBS1* transcript. Significance was calculated either between intron 5 retention, correctly spliced or exon 5 skipping bands in reference to the untreated condition (error bars: mean ± SD). Significance levels were calculated with the Mann-Whitney-U test. *p < 0.05, **p < 0.01, ***p < 0.001; n.s., not significant. N, number of independent experiments; CS, correctly spliced transcript; ES, exon skipping; IR, intron retention; kb, kilo bases.

were verified by Sanger sequencing and were confirmed to be the expected *BBS1* splice products (intron 5 retention, correctly spliced *BBS1* transcripts, or exon 5 skipping). Analyses of agarose gel electrophoreses and densitometric measurements of RT-PCR band intensities were performed with an imaging system (ChemiDoc MP imaging system and Image lab 6.0 software, Biorad, Munich, Germany).

Statistical Analysis

Data of all experiments are presented as mean ± SD. Each experiment was replicated at least three times with independently cultured and treated cells. Statistical analysis was performed with IBM SPSS Statistics software, version 25. Statistical significance was analyzed using the nonparametric Mann-Whitney U test.

SUPPLEMENTAL INFORMATION

Supplemental Information can be found online at https://doi.org/10.1016/j.omtn.2019.08.014.

AUTHOR CONTRIBUTIONS

Conceptualization, J.N.; Methodology, J.N., S.B., M.O.-L., and M.V.; Validation, S.B. and M.V.; Formal Analysis, S.B.; Investigation, S.B., M.O.-L., M.V.; Resources, J.N. and A.U.B.; Data Curation, S.B., M.V.; Writing – Original Draft Preparation, J.N. and S.B.; Writing – Review & Editing, J.N., S.B., A.U.B., and M.O.-L.; Visualization, S.B. and J.N.; Supervision, J.N.; Project Administration, J.N.; Funding Acquisition, J.N.

CONFLICTS OF INTEREST

The authors declare no competing interests.

ACKNOWLEDGMENTS

We are grateful to patients and their family for participating in this study. Furthermore, we thank Andrea Korte for support in cell culture and technical assistance and Patricia Näder for helpful discussions. The study was supported by institutional funding resources from the Faculty of Medicine and Health Science at the University of Oldenburg, Germany.

REFERENCES

1. Farag, T.I., and Teebi, A.S. (1989). High incidence of Bardet Biedl syndrome among the Bedouin. Clin. Genet. *36*, 463–464.
2. Moore, S.J., Green, J.S., Fan, Y., Bhogal, A.K., Dicks, E., Fernandez, B.A., Stefanelli, M., Murphy, C., Cramer, B.C., Dean, J.C., et al. (2005). Clinical and genetic epidemiology of Bardet-Biedl syndrome in Newfoundland: a 22-year prospective, population-based, cohort study. Am. J. Med. Genet. A. *132A*, 352–360.
3. Hjortshøj, T.D., Grønskov, K., Brøndum-Nielsen, K., and Rosenberg, T. (2009). A novel founder BBS1 mutation explains a unique high prevalence of Bardet-Biedl syndrome in the Faroe Islands. Br. J. Ophthalmol. *93*, 409–413.
4. Suspitsin, E.N., and Imyanitov, E.N. (2016). Bardet-Biedl Syndrome. Mol. Syndromol. *7*, 62–71.
5. Forsythe, E., Kenny, J., Bacchelli, C., and Beales, P.L. (2018). Managing Bardet-Biedl Syndrome-Now and in the Future. Front Pediatr. *6*, 23.
6. Mykytyn, K., Nishimura, D.Y., Searby, C.C., Shastri, M., Yen, H.J., Beck, J.S., Braun, T., Streb, L.M., Cornier, A.S., Cox, G.F., et al. (2002). Identification of the gene (BBS1) most commonly involved in Bardet-Biedl syndrome, a complex human obesity syndrome. Nat. Genet. *31*, 435–438.
7. Davis, R.E., Swiderski, R.E., Rahmouni, K., Nishimura, D.Y., Mullins, R.F., Agassandian, K., Philp, A.R., Searby, C.C., Andrews, M.P., Thompson, S., et al. (2007). A knockin mouse model of the Bardet-Biedl syndrome 1 M390R mutation has cilia defects, ventriculomegaly, retinopathy, and obesity. Proc. Natl. Acad. Sci. USA *104*, 19422–19427.
8. Schmid, F., Glaus, E., Barthelmes, D., Fliegauf, M., Gaspar, H., Nürnberg, G., Nürnberg, P., Omran, H., Berger, W., and Neidhardt, J. (2011). U1 snRNA-mediated gene therapeutic correction of splice defects caused by an exceptionally mild BBS mutation. Hum. Mutat. *32*, 815–824.
9. Bonifert, T., Gonzalez Menendez, I., Battke, F., Theurer, Y., Synofzik, M., Schöls, L., and Wissinger, B. (2016). Antisense Oligonucleotide Mediated Splice Correction of a Deep Intronic Mutation in OPA1. Mol. Ther. Nucleic Acids *5*, e390.
10. Garanto, A., Chung, D.C., Duijkers, L., Corral-Serrano, J.C., Messchaert, M., Xiao, R., Bennett, J., Vandenberghe, L.H., and Collin, R.W. (2016). In vitro and in vivo rescue of aberrant splicing in CEP290-associated LCA by antisense oligonucleotide delivery. Hum. Mol. Genet. *25*, 2552–2563.
11. Slijkerman, R.W., Vaché, C., Dona, M., García-García, G., Claustres, M., Hetterschijt, L., Peters, T.A., Hartel, B.P., Pennings, R.J., Millan, J.M., et al. (2016). Antisense Oligonucleotide-based Splice Correction for USH2A-associated Retinal Degeneration Caused by a Frequent Deep-intronic Mutation. Mol. Ther. Nucleic Acids *5*, e381.
12. Albert, S., Garanto, A., Sangermano, R., Khan, M., Bax, N.M., Hoyng, C.B., Zernant, J., Lee, W., Allikmets, R., Collin, R.W.J., and Cremers, F.P.M. (2018). Identification and Rescue of Splice Defects Caused by Two Neighboring Deep-Intronic ABCA4 Mutations Underlying Stargardt Disease. Am. J. Hum. Genet. *102*, 517–527.
13. Duijkers, L., van den Born, L.I., Neidhardt, J., Bax, N.M., Pierrache, L.H.M., Klevering, B.J., Collin, R.W.J., and Garanto, A. (2018). Antisense Oligonucleotide-Based Splicing Correction in Individuals with Leber Congenital Amaurosis due to Compound Heterozygosity for the c.2991+1655A>G Mutation in CEP290. Int. J. Mol. Sci. *19*, 19.
14. Sangermano, R., Garanto, A., Khan, M., Runhart, E.H., Bauwens, M., Bax, N.M., van den Born, L.I., Khan, M.I., Conelis, S.S., Verheij, J.B.G.M., et al. (2019). Deep-intronic ABCA4 variants explain missing heritability in Stargardt disease and allow correction of splice defects by antisense oligonucleotides. Genet. Med *21*, 1751–1760.
15. Collin, R.W., den Hollander, A.I., van der Velde-Visser, S.D., Bennicelli, J., Bennett, J., and Cremers, F.P. (2012). Antisense Oligonucleotide (AON)-based Therapy for Leber Congenital Amaurosis Caused by a Frequent Mutation in CEP290. Mol. Ther. Nucleic Acids *1*, e14.
16. Lee, Y., and Rio, D.C. (2015). Mechanisms and Regulation of Alternative Pre-mRNA Splicing. Annu. Rev. Biochem. *84*, 291–323.
17. Nilsen, T.W. (2003). The spliceosome: the most complex macromolecular machine in the cell? BioEssays *25*, 1147–1149.
18. Will, C.L., and Lührmann, R. (2011). Spliceosome structure and function. Cold Spring Harb. Perspect. Biol. *3*, a003707.
19. Hammond, S.M., and Wood, M.J. (2011). Genetic therapies for RNA mis-splicing diseases. Trends Genet. *27*, 196–205.
20. Havens, M.A., Duelli, D.M., and Hastings, M.L. (2013). Targeting RNA splicing for disease therapy. Wiley Interdiscip. Rev. RNA *4*, 247–266.
21. Berger, W., Kloeckener-Gruissem, B., and Neidhardt, J. (2010). The molecular basis of human retinal and vitreoretinal diseases. Prog. Retin. Eye Res. *29*, 335–375.
22. Glöckle, N., Kohl, S., Mohr, J., Scheurenbrand, T., Sprecher, A., Weisschuh, N., Bernd, A., Rudolph, G., Schubach, M., Poloschek, C., et al. (2014). Panel-based next generation sequencing as a reliable and efficient technique to detect mutations in unselected patients with retinal dystrophies. Eur. J. Hum. Genet. *22*, 99–104.
23. Krawczak, M., Thomas, N.S., Hundrieser, B., Mort, M., Wittig, M., Hampe, J., and Cooper, D.N. (2007). Single base-pair substitutions in exon-intron junctions of human genes: nature, distribution, and consequences for mRNA splicing. Hum. Mutat. *28*, 150–158.
24. Wang, G.S., and Cooper, T.A. (2007). Splicing in disease: disruption of the splicing code and the decoding machinery. Nat. Rev. Genet. *8*, 749–761.
25. Cooper, T.A., Wan, L., and Dreyfuss, G. (2009). RNA and disease. Cell *136*, 777–793.

26. Pinotti, M., Balestra, D., Rizzotto, L., Maestri, I., Pagani, F., and Bernardi, F. (2009). Rescue of coagulation factor VII function by the U1+5A snRNA. Blood *113*, 6461–6464.

27. Tanner, G., Glaus, E., Barthelmes, D., Ader, M., Fleischhauer, J., Pagani, F., Berger, W., and Neidhardt, J. (2009). Therapeutic strategy to rescue mutation-induced exon skipping in rhodopsin by adaptation of U1 snRNA. Hum. Mutat. *30*, 255–263.

28. Glaus, E., Schmid, F., Da Costa, R., Berger, W., and Neidhardt, J. (2011). Gene therapeutic approach using mutation-adapted U1 snRNA to correct a RPGR splice defect in patient-derived cells. Mol. Ther. *19*, 936–941.

29. Pinotti, M., Rizzotto, L., Balestra, D., Lewandowska, M.A., Cavallari, N., Marchetti, G., Bernardi, F., and Pagani, F. (2008). U1-snRNA-mediated rescue of mRNA processing in severe factor VII deficiency. Blood *111*, 2681–2684.

30. Roca, X., and Krainer, A.R. (2009). Recognition of atypical 5′ splice sites by shifted base-pairing to U1 snRNA. Nat. Struct. Mol. Biol. *16*, 176–182.

31. Hartmann, L., Neveling, K., Borkens, S., Schneider, H., Freund, M., Grassman, E., Theiss, S., Wawer, A., Burdach, S., Auerbach, A.D., et al. (2010). Correct mRNA processing at a mutant TT splice donor in FANCC ameliorates the clinical phenotype in patients and is enhanced by delivery of suppressor U1 snRNAs. Am. J. Hum. Genet. *87*, 480–493.

32. Sánchez-Alcudia, R., Pérez, B., Pérez-Cerdá, C., Ugarte, M., and Desviat, L.R. (2011). Overexpression of adapted U1snRNA in patients' cells to correct a 5′ splice site mutation in propionic acidemia. Mol. Genet. Metab. *102*, 134–138.

33. Fernandez Alanis, E., Pinotti, M., Dal Mas, A., Balestra, D., Cavallari, N., Rogalska, M.E., Bernardi, F., and Pagani, F. (2012). An exon-specific U1 small nuclear RNA (snRNA) strategy to correct splicing defects. Hum. Mol. Genet. *21*, 2389–2398.

34. Balestra, D., Faella, A., Margaritis, P., Cavallari, N., Pagani, F., Bernardi, F., Arruda, V.R., and Pinotti, M. (2014). An engineered U1 small nuclear RNA rescues splicing defective coagulation F7 gene expression in mice. J. Thromb. Haemost. *12*, 177–185.

35. Schmid, F., Hiller, T., Korner, G., Glaus, E., Berger, W., and Neidhardt, J. (2013). A gene therapeutic approach to correct splice defects with modified U1 and U6 snRNPs. Hum. Gene Ther. *24*, 97–104.

36. Gerard, X., Garanto, A., Rozet, J.M., and Collin, R.W. (2016). Antisense Oligonucleotide Therapy for Inherited Retinal Dystrophies. Adv. Exp. Med. Biol. *854*, 517–524.

37. Garanto, A., van der Velde-Visser, S.D., Cremers, F.P.M., and Collin, R.W.J. (2018). Antisense Oligonucleotide-Based Splice Correction of a Deep-Intronic Mutation in CHM Underlying Choroideremia. Adv. Exp. Med. Biol. *1074*, 83–89.

38. Wormser, O., Gradstein, L., Yogev, Y., Perez, Y., Kadir, R., Goliand, I., Sadka, Y., El Riati, S., Flusser, H., Nachmias, D., et al. (2019). SCAPER localizes to primary cilia and its mutation affects cilia length, causing Bardet-Biedl syndrome. Eur. J. Hum. Genet. *27*, 928–940.

39. Beltinger, C., Saragovi, H.U., Smith, R.M., LeSauteur, L., Shah, N., DeDionisio, L., Christensen, L., Raible, A., Jarett, L., and Gewirtz, A.M. (1995). Binding, uptake, and intracellular trafficking of phosphorothioate-modified oligodeoxynucleotides. J. Clin. Invest. *95*, 1814–1823.

40. McKay, R.A., Miraglia, L.J., Cummins, L.L., Owens, S.R., Sasmor, H., and Dean, N.M. (1999). Characterization of a potent and specific class of antisense oligonucleotide inhibitor of human protein kinase C-alpha expression. J. Biol. Chem. *274*, 1715–1722.

41. Geary, R.S., Watanabe, T.A., Truong, L., Freier, S., Lesnik, E.A., Sioufi, N.B., Sasmor, H., Manoharan, M., and Levin, A.A. (2001). Pharmacokinetic properties of 2′-O-(2-methoxyethyl)-modified oligonucleotide analogs in rats. J. Pharmacol. Exp. Ther. *296*, 890–897.

42. Eckstein, F. (2014). Phosphorothioates, essential components of therapeutic oligonucleotides. Nucleic Acid Ther. *24*, 374–387.

43. Evers, M.M., Toonen, L.J., and van Roon-Mom, W.M. (2015). Antisense oligonucleotides in therapy for neurodegenerative disorders. Adv. Drug Deliv. Rev. *87*, 90–103.

44. Brenner, S., and Malech, H.L. (2003). Current developments in the design of oncoretrovirus and lentivirus vector systems for hematopoietic cell gene therapy. Biochim. Biophys. Acta *1640*, 1–24.

45. Werner, M., Kraunus, J., Baum, C., and Brocker, T. (2004). B-cell-specific transgene expression using a self-inactivating retroviral vector with human CD19 promoter and viral post-transcriptional regulatory element. Gene Ther. *11*, 992–1000.

46. Lund, E., and Dahlberg, J.E. (1984). True genes for human U1 small nuclear RNA. Copy number, polymorphism, and methylation. J. Biol. Chem. *259*, 2013–2021.

OMTN, Volume *18*

Supplemental Information

Combining Engineered U1 snRNA and Antisense Oligonucleotides to Improve the Treatment of a *BBS1* Splice Site Mutation

Saskia Breuel, Mariann Vorm, Anja U. Bräuer, Marta Owczarek-Lipska, and John Neidhardt

Supplemental Material

Combining designed U1 snRNA and antisense oligonucleotides to improve the treatment of a *BBS1* splice site mutation.

Saskia Breuel[1], Mariann Vorm[1], Anja U. Bräuer[2,3], Marta Owczarek-Lipska[1], John Neidhardt[1,3,4,]*

Figure S1:

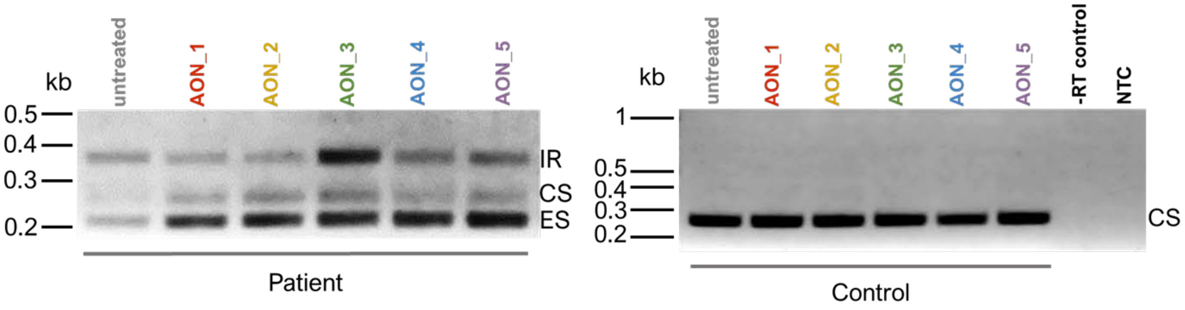

Fig. S1: Verification of AON effects during the apoptosis assay. Cells were lyzed and RT-PCR reactions were performed after the apoptosis assays. AON concentrations were 36 nM. The efficacy of the tested AONs to reduce intron 5 retention in the patient samples resembles the results shown in Fig. 3. The AON treatment did not induce splice defects in control cells. NTC: none template control; –RT control: PCR control reaction using a template with RNA but no reverse transcriptase was added during cDNA-synthesis. CS: correctly spliced transcript, ES: exon skipping, IR: intron retention, kb: kilo bases.
